# The Study of Evaluation and Rehabilitation of Patients With Different Cognitive Impairment Phases Based on Virtual Reality and EEG

**DOI:** 10.3389/fnagi.2018.00088

**Published:** 2018-04-03

**Authors:** Dong Wen, Xifa Lan, Yanhong Zhou, Guolin Li, Sheng-Hsiou Hsu, Tzyy-Ping Jung

**Affiliations:** ^1^Department of Software Engineering, School of Information Science and Engineering, Yanshan University, Qinhuangdao, China; ^2^The Key Laboratory for Computer Virtual Technology and System Integration of Hebei Province, Yanshan University, Qinhuangdao, China; ^3^Department of Neurology, First Hospital of Qinhuangdao, Qinhuangdao, China; ^4^Department of Computer Science and Technology, School of Mathematics and Information Science and Technology, Hebei Normal University of Science and Technology, Qinhuangdao, China; ^5^Swartz Center for Computational Neuroscience, University of California, San Diego, San Diego, CA, United States

**Keywords:** evaluation, rehabilitation, patients with different cognitive impairment phases, virtual reality, EEG

## Introduction

The evaluation and rehabilitation (EAR) of patients with different cognitive impairment phases (PDCIP), including subjective cognitive decline (SCD) (Jessen et al., 2014; Innes et al., 2016a,b, 2017), mild cognitive impairment (MCI) (Weniger et al., 2011) and Alzheimer's disease (AD) (Serino et al., 2015), become a rapidly growing research field. Virtual reality (VR) has been reported to re-activate and/or improve multiple cortical functions (Baumann et al., 2003; Lin et al., 2008; Schedlbauer et al., 2014; Carrieri et al., 2016) and help optimize the coding efficiency of the sensory cortex (Ansuini et al., 2006; Keller et al., 2012; Ravassard et al., 2013; Schindler and Bartels, 2013; Sofroniew et al., 2015). Therefore, many researchers have started applying the VR technology to the EAR of PDCIP (Buss, 2009), including spatial memory (Allison et al., 2016), episodic memory (Valladares-Rodriguez et al., 2017), activities of daily living (Seo et al., 2017), language (Montenegro and Argyriou, 2017), executive function (Tost et al., 2014), short-term and working memory (Burdea et al., 2013), attention (Kalová et al., 2005), movement and balance (McEwen et al., 2014), and outdoor activities (Van Schaik et al., 2008). For EAR of PDCIP, the advantages of using VR over conventional approaches, such as electroencephalogram (EEG), functional magnetic resonance imaging (fMRI), and neuropsychological tasks, have been reported (Tarnanas et al., 2014a,b, 2015a). However, these studies did not record EEG during the course of evaluation and training within a VR environment, nor took full advantages of the EEG recordings to objectively explore the brain states of PDCIP in (near) real time, despite the combination of VR and EEG has been used in the EAR of other diseases, including stroke (I Badia et al., 2013; Lechner et al., 2014; Vourvopoulos and I Badia, 2016), paraplegic (Donati et al., 2016), autism (Amaral et al., 2017), attention deficit hyperactivity disorder (Rohani and Puthusserypady, 2015) and so on. The neuroimaging research for the EAR of PDCIP within a VR environment is still in its infancy, and more works need to be done before conclusions can be confidently drawn.

This study will review the literature related to the EAR of PDCIP from the perspectives of VR and EEG, and discuss the potential advantages of the combined use of VR and EEG. It is expected that the analysis may provide useful suggestions to the field of the EAR of PDCIP.

## The ear of PDCIP based on VR

### Spatial memory impairments

Spatial memory impairment is a common symptom in PDCIP (Nedelska et al., 2012; Vlček and Laczó, 2014), and many studies have explored the EAR for the spatial memory of PDCIP using VR (Bormans et al., 2016; Tu et al., 2017). For example, Virtual Reality for Early Detection of Dementia (VReDD) including VR Practice, VR Park and VR Games was developed to assess spatial memory of early dementia patients (Shamsuddin et al., 2011, 2012). Caffò and colleagues also showed that the spatial memory impairments of amnestic MCI (aMCI) patients could be identified by a reorientation task in VR (Caffò et al., 2012).

In a virtual maze, many studies showed that the spatial orientation ability of AD patients was poorer compared to normal control (NC) (Morganti et al., 2013). The aMCI patients also performed poorly in two VR tasks (Weniger et al., 2011) and a virtual radial arm maze (Lee et al., 2014; Migo et al., 2016). Furthermore, preclinical AD patients were defective in a VR path-finding and route-learning task (Allison et al., 2016).

Recently, Zygouris et al. (2017), proposed a virtual supermarket for screening MCI from the elderly (Zygouris et al., 2017), and could be used to objectively evaluate the spatial-orientation ability of AD patients based on the integrity of the retrosplenial cortex (Tu et al., 2015). There were also different levels of impairments in judgments of allocentric and egocentric heading direction between patients with the behavioral variant fronto-temporal dementia and AD (Tu et al., 2017).

Applications of virtual buildings and rooms in VR can both evaluate and rehabilitate the spatial memory in PDCIP. In evaluation, the wayfinding and navigation ability of AD patients can be assessed based on a virtual hospital (Jiang and Li, 2007), virtual auditorium (Lange et al., 2007), virtual city (Zakzanis et al., 2009), virtual buildings (Cushman et al., 2008), and virtual environments of senior residential buildings (Davis and Ohman, 2016). In addition, Pengas et al. found the relationship between topographic memory capacity and regional neurodegeneration in AD patients using a virtual route-learning test (Pengas et al., 2012). For MCI patients, a virtual road-navigation task was used to evaluate visual-spatial memory (Lesk et al., 2014), and a virtual room-location-searching task was used to detect the impairments of egocentric and allocentric spatial-navigation (Serino et al., 2015). In rehabilitation, virtual memory palaces could improve the living quality and memory of AD patients (Bormans et al., 2016), and a VR building-navigation game could enhance their driving skills and daily cognitive abilities (White and Moussavi, 2016).

### Episodic memory impairment

An episodic memory impairment, which often co-exists with the spatial memory impairment in aMCI and AD patients, is an important indicator of PDCIP (Bellassen et al., 2012). VR has been used to evaluate the episodic memory of PDCIP (Valladares-Rodriguez et al., 2017). For AD patients, the virtual alleys could be utilized to evaluate and predict their the temporal order memory in the episodic memory (Bellassen et al., 2012), and the virtual living scene could be used to evaluate their episodic memory and executive function (Sauzéon et al., 2016). The impairments of episodic and spatial memory often co-exist in patients with aMCI and AD. Plancher et al. showed that active and passive encoding in a virtual driving task could help distinguish the patients from NC (Plancher et al., 2012). Serino and Riva also reported that unconsciousness anosognosia, which might be caused by episodic and spatial memory disorders, could be a biomarker of AD patients (Serino and Riva, 2017).

### Other cognitive impairments

#### Daily living tasks

Virtual-reality kitchen (VRK) is a popular research tool for the EAR of PDCIP. For evaluation, the impairments of instrumental activities of AD patients could be assessed by the coffee-making task (Allain et al., 2014) and the cooking task (Vallejo et al., 2017), in which the patients required more time to complete compared with NC. In addition, aMCI patients could be evaluated by a virtual apartment fire-evacuation drill (Tarnanas et al., 2015b) and kinematic measures in some virtual tasks including withdrawing cash and taking a bus (Seo et al., 2017). Virtual supermarket is another commonly used tool, which meets the standard of the neuropsychological examination utilized in the diagnosis of MCI (Tsolaki et al., 2015), and can be used to remotely evaluate MCI (Zygouris et al., 2017). For rehabilitation, studies have shown that AD patients could improve their good performance by receiving training with daily cooking activities in a Dual-Modal VRK (Yamaguchi et al., 2012) and a kitchen and cooking virtual game (Manera et al., 2015). The daily living ability of AD patients could also be improved by practicing some virtual tasks (Hofmann et al., 2003).

#### Language memory and expression

AD patients could be diagnosed based on their memory of verbal material and spatial scenery within VR (Widmann et al., 2012). Screening tests within virtual-room game include language expression and understanding (Montenegro and Argyriou, 2015, 2017). In addition, interviews with patients in VR environments could be used to evaluate frontotemporal dementia (Mendez et al., 2015).

#### Executive function, memory and attention

The VR tools, including a virtual action-planning supermarket (Werner et al., 2009), virtual store (Yeh et al., 2012), virtual reality day-out task (Tarnanas et al., 2013, 2014a) and 3D family virtual environment and task (Tost et al., 2014), are often used in the assessment of executive functions, memory and attention capabilities of PDCIP. In addition, VR could be utilized to evaluate the attention of AD patients (Kalová et al., 2005), rehabilitate the short-term and working memory of patients with advanced dementia (Burdea et al., 2013), enhance the objective memory of old people with questionable dementia (Man et al., 2012), and improve the attention of MCI and dementia patients (Manera et al., 2016).

#### Movement and balance

Studies suggested that VR training might improve the balance and mobility of dementia patients (McEwen et al., 2014) and recover the motor and postural abilities of the MCI patients (Bourrelier et al., 2016).

#### Outdoor activities

Outdoor activities within VR can help evaluate and train patients with dementia. VR could be used to simulate the environment of outdoor activities and thereby improve the frequency of their outdoor activities (Blackman et al., 2007), to enable the patients to plan and test outdoor design (Blackman et al., 2003), and to detect and improve their ability of functioning outdoors (Van Schaik et al., 2008). In addition, the virtual environment of a large outdoor park could be utilized to evaluate the navigation and control ability of the patients (Flynn et al., 2003).

### Current challenges

Although the EAR of PDCIP based on VR has shown preliminary achievements, current methods used to test the effectiveness of the EAR are mainly confined to the assessments of neuropsychological scales, subjective judgments of experimenters and researchers, and qualitative descriptions and feedback from patients and their dependents. These examination methods might not lead to accurate and timely evaluations, nor provide the scientific evidence for the effectiveness of VR during and after training. Hence, there is a need for objective and quantitative evaluation methods, ideally in near real-time, for the diagnosis and rehabilitation within VR.

## The status of combining VR and EEG technology and its application in the EAR of PDCIP

### The research value and status of VR and EEG

EEG is often used in clinical evaluation and detection of neurological diseases. Compared to other noninvasive neuroimaging technologies such as fMRI, EEG provides a direct, real-time measurement of brain activity with high temporal resolution. In addition, EEG recording devices can be portable and relatively low cost, enabling real-world applications. The combined use of the EEG within VR (VR-EEG) may provide substantial benefits to EAR. On one hand, EEG can simultaneously record a person's brain activity and brain state in near real-time during VR training. On the other hand, VR can be used to provide a scenario close to person's life, in which the brain state can be evaluated based on EEG. Hence, VR-EEG might resolve the issues of subjective and unpunctual evaluation in the EAR and can quantitatively evaluate the effectiveness of VR training in near real-time.

Recently, studies of VR-EEG technology have begun to take shape. For example, some studies employed brain-computer interface (BCI) technologies within VR, including the steady-state visual evoked potential BCI for rehabilitating stroke (Lechner et al., 2014), overcoming the limitations of refresh frequency of a regular monitor (Calore et al., 2014), and improving the user engagement (Koo et al., 2015), the motor-imagery BCI for stroke rehabilitation (I Badia et al., 2013; Vourvopoulos and I Badia, 2016) and paraplegic patients (Donati et al., 2016), and the P300 BCI for training normal aging subjects (de Tommaso et al., 2016) and patients with autism disorder (Amaral et al., 2017) and attention deficit hyperactivity disorder (Rohani and Puthusserypady, 2015).

### The VR and EEG for the EAR of PDCIP

Recently, several studies added the measurements of EEG into VR training and evaluation of PDCIP, and compared the performance of VR and EEG (Tarnanas et al., 2014a,b, 2015a). For example, VR day-out task (VR-DOT) was used to train MCI patients, whose brain states were evaluated by EEG, fMRI and neuropsychological scales before and after the training, and the result suggested that the performance of VR-DOT correlated strongly with event-related potentials of cortical thickness (Tarnanas et al., 2014a). In addition, the virtual action-planning museum and EEG were also used in assessing spatial navigation, prospective memory and executive function of the MCI patients, the results also suggested using VR over EEG (Tarnanas et al., 2014b, 2015a). However, these studies did not measure the EEG activities of the patients during their training or rehabilitation sessions to assess the effectiveness of training and provide feedback to the patients. In other words, they still used an open-loop therapeutic method, and the true value of the combined use of VR and EEG has not been implemented or explored for the EAR of PDCIP.

## Future direction of EAR for PDCIP based on VR and EEG

While achievements have been made in the EAR for the MCI and AD patients based on VR, the VR-based EAR for patients with SCD, a preclinical state of MCI and AD, has not yet started. The evaluation of SCD patients is mainly limited to Cerebrospinal fluid, fMRI and EEG methods (Sun et al., 2015; Zhou et al., 2016). The rehabilitation of the SCD is confined to meditation, music therapy (Innes et al., 2016b, 2017), and mindfulness training (Smart et al., 2016). The studies have achieved promising results, however some subjects in these studies were unable to complete the experiments, the sample size of these studies was fairly small, and their follow-up period was short. In the future, for the EAR of SCD, MCI, and AD patients, meditation, music and mindfulness training will can be incorporated into immersive VR environments to engage the patients and thereby improve the effectiveness of training. In addition, brain activities of the patients can be measured during training to objectively and quantitatively evaluate the progress of the rehabilitation, which could motivate the patients to continue the rehabilitation process. As mentioned in the literature (Anguera et al., 2013; de Tommaso et al., 2016), one can use VR for the EAR of PDCIP and simultaneously collect EEG signals of patients to assess the effectiveness of rehabilitation. Alternatively, EEG signals of PDCIP can be used to control/interact with virtual characters in VR to make the training more interactive and even entertaining to improve the effects of the EAR. In addition, fMRI, near infrared spectroscopy, and cerebral oxygen metabolism markers can also be used to evaluate the PDCIP before and after the training based on VR and EEG.

## Conclusion

In conclusion, this study reviewed the recent literature of the EAR of PDCIP based on VR. Although these studies obtained promising results, more work needs to be done before conclusions can be confidently drawn. We also suggest collecting EEG activities of the patients before, during, and after the training to assess the bio-markers of neuroplasticity and monitor the progress of the rehabilitation. The ultimate goal is to create and evaluate a closed-loop EAR system for PDCIP. As we all know, without any intervention, the patients with cognitive impairments would have a poor prognosis such as rapid cognitive decline as they age. However, the neuroplasticity provides a possibility for intervention. Therefore, some new approaches, such as TMS and effective cognitive training including VR, could be developed to intervene the decline of metabolic efficiency and neuroplasticity with age. In particular, many studies have shown that the cognitive training in VR could enhance the neuroplasticity of the brain (Johnson et al., 1998; Anopas and Wongsawat, 2014; Robles-García et al., 2016; Teo et al., 2016), and help repair damaged brain circuits (Subramanian and Prasanna, 2017; Yang et al., 2017). In consequence, we postulate that the cognitive training using VR and EEG can intervene effectively and help improve the cognitive functions of the patients in this paper.

## Author contributions

DW designed the study and wrote this paper, DW, XL, and YZ analyzed literature, GL and S-HH revised this paper, T-PJ designed the study and revised this paper.

### Conflict of interest statement

The authors declare that the research was conducted in the absence of any commercial or financial relationships that could be construed as a potential conflict of interest.
